# Efficiency of Machine Learning Algorithms for the Determination of Macrovesicular Steatosis in Frozen Sections Stained with Sudan to Evaluate the Quality of the Graft in Liver Transplantation

**DOI:** 10.3390/s21061993

**Published:** 2021-03-12

**Authors:** Fernando Pérez-Sanz, Miriam Riquelme-Pérez, Enrique Martínez-Barba, Jesús de la Peña-Moral, Alejandro Salazar Nicolás, Marina Carpes-Ruiz, Angel Esteban-Gil, María Del Carmen Legaz-García, María Antonia Parreño-González, Pablo Ramírez, Carlos M. Martínez

**Affiliations:** 1 Biomedical Informatics & Bioinformatics Service, Institute for Biomedical Research of Murcia (IMIB), 30120 Murcia, Spain; angel.esteban@ffis.es (A.E.-G.); mcarmen.legaz@ffis.es (M.D.C.L.-G.); maria.parreno@ffis.es (M.A.P.-G.); 2 CNRS-CEA, University Paris-Saclay, MIRCen, 92265 Paris, France; miriam.riquelme-perez@cea.fr; 3 Pathology Service, University Clinical Hospital Virgen de la Arrixaca-Biomedical Research Institute of Murcia (IMIB), 30120 Murcia, Spain; enrique.martinez4@carm.es (E.M.-B.); jesus@biopathocid.com (J.d.l.P.-M.); alejandro.salazar@um.es (A.S.N.); 4 Experimental Pathology Service, Institute for Biomedical Research of Murcia (IMIB), 30120 Murcia, Spain; marina.carpes@imib.es (M.C.-R.); carlos.martinez@imib.es (C.M.M.); 5 General and Digestive Surgery Service, University Clinical Hospital Virgen de la Arrixaca-Biomedical Research Institute of Murcia (IMIB), 30120 Murcia, Spain

**Keywords:** liver transplantation, sudan stain, machine learning, computer vision, macrovesicular steatosis

## Abstract

Liver transplantation is the only curative treatment option in patients diagnosed with end-stage liver disease. The low availability of organs demands an accurate selection procedure based on histological analysis, in order to evaluate the allograft. This assessment, traditionally carried out by a pathologist, is not exempt from subjectivity. In this sense, new tools based on machine learning and artificial vision are continuously being developed for the analysis of medical images of different typologies. Accordingly, in this work, we develop a computer vision-based application for the fast and automatic objective quantification of macrovesicular steatosis in histopathological liver section slides stained with Sudan stain. For this purpose, digital microscopy images were used to obtain thousands of feature vectors based on the RGB and CIE L*a*b* pixel values. These vectors, under a supervised process, were labelled as fat vacuole or non-fat vacuole, and a set of classifiers based on different algorithms were trained, accordingly. The results obtained showed an overall high accuracy for all classifiers (>0.99) with a sensitivity between 0.844 and 1, together with a specificity >0.99. In relation to their speed when classifying images, KNN and Naïve Bayes were substantially faster than other classification algorithms. Sudan stain is a convenient technique for evaluating ME in pre-transplant liver biopsies, providing reliable contrast and facilitating fast and accurate quantification through the machine learning algorithms tested.

## 1. Introduction

Liver transplantation is the unique curative option for those patients with end-stage liver disease and acute liver failure. The progressive demand for transplants and the limited number of available organs have led to modification of the scoring systems used to assess post-transplant complications, in order to include livers excluded according to the older systems—such as organs from cardiac death (CDC), and HIV- and/or hepatitis C-infected patients—into the donor pool [1]. However, this fact also implies that the analytic procedures to evaluate the suitability of the grafts must also be more accurate. Therefore, histological analysis to assess the quality of the allografts is crucial to prevent graft dysfunction or secondary rejection.

As there is a strong relationship between the ischemia time—defined as the time from graft clamping to pre-implant reperfusion—and the risk of graft failure [2], this analysis must be assessed as soon as possible, in order to prevent severe liver damage. Thus, the use of hematoxylin and eosin (H & E) stained sections from frozen representative graft samples, instead of routine-processed ones, is a time effective alternative for this determination, as it allows for a rapid histological examination [3]. During histological analysis, several parameters should be assessed. One such parameter is the presence of big intracytoplasmic lipid droplets—macrovesicular steatosis (ME)—a feature which has shown predictive value for graft dysfunction [4]. Thus, livers with <30% ME are generally considered acceptable for transplantation [5,6], although this value varies by institution and can be increased up to 60% [7]. Nevertheless, this criteria is based on subjective evaluation by an experienced pathologist, which is strongly observation-dependent, non-reproducible, and challenging, even in experienced hands [8,9]. Additionally, the main limitation of the use of H&E frozen-stained sections is the risk of the underestimation of ME, due to the presence of artifacts (e.g., water droplets) during the sampling procedure [3,10]. Thus, the development of alternative technical and analytic procedures for staining and examining representative frozen graft sections, which allow for the establishment of an objective ME value in the shortest possible time, should be key to ensuring the viability of the transplant.

The development of image analysis tools based on machine learning algorithms for histopathologic analysis is a extremely helpful application of computational biology, helping pathologists to establish exact and accurate diagnoses. In this sense, several image analysis algorithms to determine the degree of ME of the graft have been developed, in terms of the determination of cross-sectional surface area of lipid droplets (LD) [11], the determination of this area using a pre-determined cut-off LD size [12,13], and the use of LD-induced nuclear dislocation of hepatocytes as methods to improve the algorithms [14], or even the use of a four-stage approach, including k-means clustering and image manipulation algorithms to detect fat areas [14]. Nevertheless, the main limitation of all of these applications is that their analytic procedures are based on H&E stained sections, which may induce an underestimation of the value [3,15]. Although several deep learning segmentation algorithms [10,16,17] and supervised machine learning procedures [18] have been proposed to improve the accuracy of such algorithms in classifying intracellular fat vacuoles—identified as white spaces in H&E sections—the use of these applications is still not fully standardized. Other solutions have been proposed, such as the assessment of liver steatosis by liver texture analysis, a macroscopic determination which uses machine learning to speed up and automate the process [19]; a non-microscopic parameter which is still under validation. Thus, the use of alternative and specific staining procedures, which allow for the development of simpler, quicker, and specific image analysis algorithms to determine ME in biopsies, is crucial in establishing an objective diagnosis in the shortest possible time.

Consequently, the use of specific fat-staining procedures is the first stage to overcome the limitations of H&E staining. Thus, the Sudan staining procedure may provide a good alternative, as it is a fat-specific, quick, and easy stain procedure which is performed only on frozen sections, showing higher sensitivity for the detection of steatosis, compared to H & E [15]. Additionally, and due to the limited time available to perform this analysis prior to the surgery, it is also necessary to optimize the analytic speed, the accuracy, and the computational cost of the specific image analysis technique. Thus, the time required to obtain the image set, the quality and the size of the images, and the time needed to obtain the final result are variables which must also be optimized. Therefore, the aim of this report is to develop an image analysis application, based on machine learning algorithms, to automatically determine the percentage of ME in a representative sample from a donor liver using Sudan stained frozen sections, which may allow for optimized accuracy with the lowest image quality possible and, thus, lower computational costs (by using the minimum system requirements possible, even allowing for the use of an on-line application for the analysis), through pre-existing and validated machine/deep learning algorithms.

## 2. Results

### 2.1. Train and Test Model

**Figure 1:**
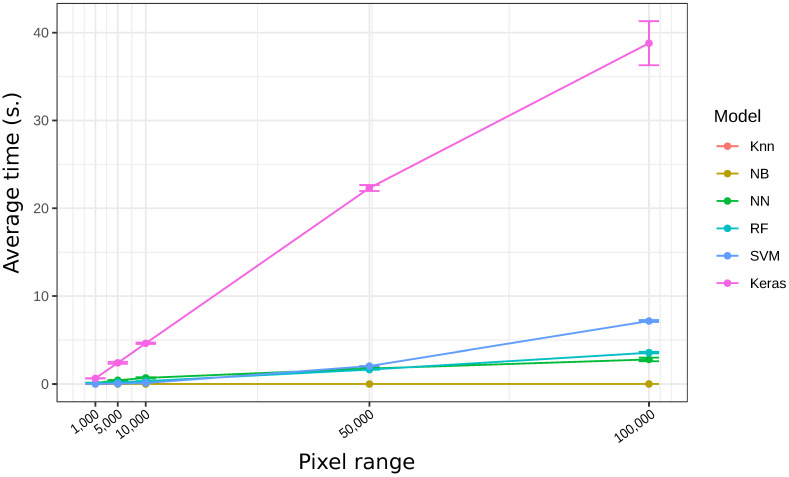
Average training time for each algorithm (in s), according with the proposed range of pixels selected.

The application was able to easily differentiate specific fat staining from artifacts related to the staining procedure. The average time that each algorithm takes to be trained is a function of the number of pixels involved in the training, which ranged from 1000 to 100,000 pixels for each model (Figure 1). Our results showed that using images of Sudan stained pre-transplant human donor liver sections, the training time using Keras increased drastically, from 0.64 s with 1000 pixels to 38 s with 100,000 pixels (Table 1). On the other hand, Naïve Bayes and KNN were the fastest algorithms in training stage, with the longest times of 0.011 and 0.006 s with 100,000 pixels, respectively.

**Figure 2:**
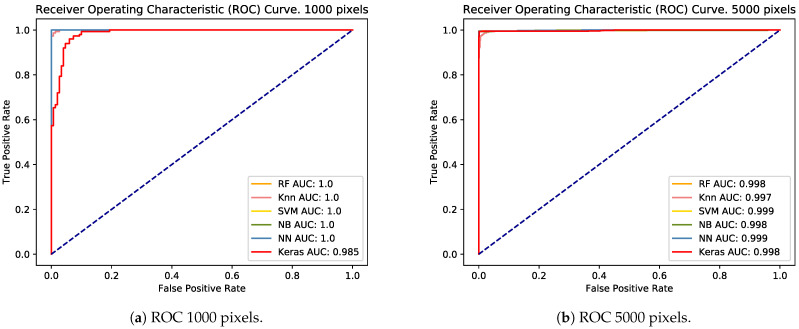

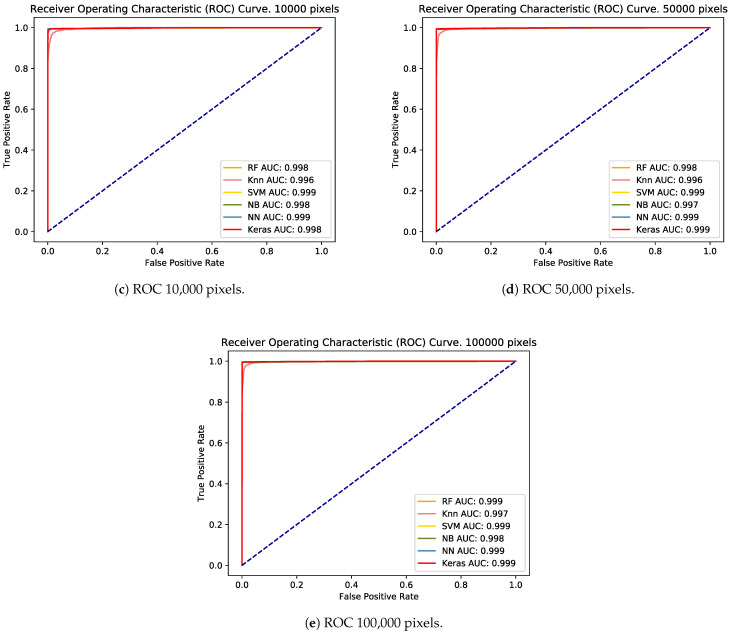
ROC curves with the AUCs of all classifiers for each training/testing data set from 1000 (**a**) to 100,000 (**e**) pixels.

The trained classifiers were tested with a test data set, comprised of 30% of the sample corresponding to each set of pixels. Overall, the performance achieved by the classifiers with different sets of pixels was close to 1. Thus, the analysis of the area under the curve (AUC) obtained from the different ROC curves was also close to 1 (Figure 2a–e). Only Keras with 0.98 had an AUC below 0.99 for the 1000-pixel proposed model (Table 1, Figure 2a).

### 2.2. Classification Time

**Figure 3:**
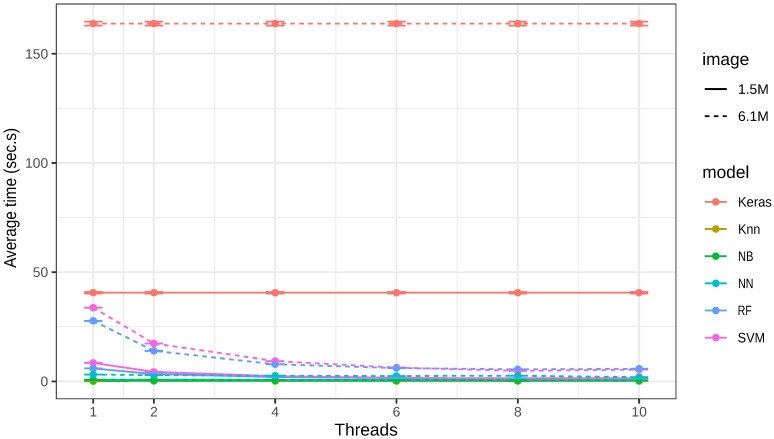
Classification time (in s) of each model used.

The time taken to classify one image differed widely, depending on the classifier and the image size (from 1.5 to 6.1 MB; Table 2 and Figure 3). Regardless of the image size, KNN and NB were the fastest algorithms, being slightly affected by both the number of threads and the image size. On the other hand, SVM and RF were the slowest algorithms, which were strongly affected by the size of the image and the number of processing threads. As Keras handles the number of threads independently, due to its implementation, it always involves all threads of the processor. Thus, the average classification time with this algorithm was always the same (Figure 3).

### 2.3. Classification Validation

**Figure 4:**
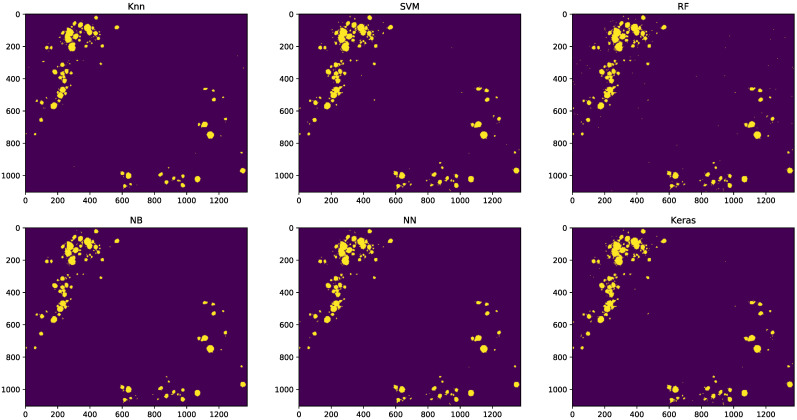
Results of image classification for each classifier.

Images classified by every model were compared with the same image manually classified by an expert pathologist (Figure 4).

Based on this comparison between images, we have calculated the accuracy (ratio of well-classified pixels to total pixels; Equation (2)), sensitivity (ratio of detected fatty vacuole pixels to total fatty vacuole pixels; Equation (3)), and specificity (ratio of non-fatty vacuole pixels detected to total non-fatty pixels; Equation (4)).

In all cases (Table 3), the accuracy, sensitivity, and specificity values were above 0.95; except for KNN, whose sensitivity was 0.844. These values were consistent with those obtained from evaluating classifiers with the train/test data sets, as shown by the ROC curves.

## 3. Discussion

Our goal was to develop an application which is able to establish an objective and reliable value of macrovesicular steatosis from representative sections of pre-transplant liver donor biopsies stained with Sudan—a fat-specific staining procedure—with minimum requirements, in terms of image quality and processing time. For this purpose, we tested several classification machine learning algorithms, in order to determine which algorithm is the most suitable for application. To the best of our knowledge, this is the first report in which several machine learning algorithms were tested for the automatic analysis of fat-specific dye stained sections for biomedical purposes. Moreover, we developed a graphical user interface (GUI) implementing the algorithms discussed in this work. It also allows for the the training of new models, based on the same algorithms. The development framework used was *Shiny*—a web development framework based on R—which allows for near-native integration of all Python code necessary for generating the models and analyzing the images. The simple and intuitive design makes it easy for the end-user to quickly quantify steatosis.

Although the number of potential donors for liver transplant has increased, the number of canceled transplantations due to a high grade of ME have also risen [20]. As this parameter still must undergo a subjective evaluation, the possibility of an error of criteria cannot be excluded; even in the case of analysis by an expert pathologist. Liver transplantation is an extremely complex surgery, the success of which depends on the time consumed between organ extraction from the donor and its reperfusion into the patient. Thus, the intraoperative histopathologic evaluation—which usually involves sampling, sectioning, staining, examination, and diagnosis—must be assessed in less than 30–45 min [21]. As the fastest fixation and paraffin embedding procedures usually require 3–4 h, the use of frozen samples is mandatory in this case. H & E is usually the standard procedure for general evaluation, which is easy and quick to perform and usually provides a good contrast to evaluate many parameters used to establish the quality of the graft for transplant. Nevertheless, this procedure does not stain fat, and the possibility of overestimating ME due to artifacts produced during the processing of the frozen biopsies (e.g., water droplets, holes, and so on) may be not discarded [3,10]. Taking this into account, coupled with the fact that ME determination is strongly observation-dependent, we find that the risk of error of judgement can increase significantly, with severe consequences, regardless of the final decision.

Thus, improvement of the staining procedure and the accuracy of the steatosis determination, by transforming an estimated determination into a quantitative one, in the shortest time possible may allow for a drastically diminished possibility of error, an increase in the number of viable organs, and the establishment of more accurate outcomes, in terms of viability of the graft.

As the use of frozen sections to the immediate diagnosis is mandatory, our first goal was to use an alternative staining procedure, which may replace the H & E stain and allow for fat to be stained specifically. To this end, we decided to use the Sudan stain, as it can be performed on frozen sections, is a fast and easy stain procedure, and is fat-specific, making it chromatically easy to differentiate fat from non-fat structures. A possible disadvantage of this stain procedure is that the value of steatosis can be overestimated by direct examination, especially when the analysis depends on inexperienced pathologists. We did not observe significant variations in ME values during the validation process, probably due to the use of two experienced pathologists specialists in liver transplantation.

Once we had solved this problem, our next issue was to determine which is the best machine learning algorithm for use in the development of our analytic tool. As all reported image analysis tools have been based on the analysis of H & E stained sections [11,12,13,14], these algorithms are focused on the measurement of numerous parameters, which try to differentiate structures (i.e., fat vacuoles vs. non-fat vacuoles and unspecific structures) with similar shape and color (i.e., round unstained/white structures). As there have been no previous reports considering the use of Sudan stain for the automatic determination of ME, we decided to use six of the most-used algorithms for image analysis [22], in order to determine which is the best option—in terms of efficacy and time—for use in a new and specific image system based on Sudan-stained section analysis. Additionally, we took into account the time used for the analysis, as this parameter should not be extended, in order to assure the efficiency of the procedure. Thus, the use of high-resolution scanned images may be not useful in this particular case, as the time required to obtain and process such images (which are near 1 GB in size each) may be not applicable to study one parameter, which must be objectively determined in 5–10 min at maximum. For this reason, our tool does not currently use whole slide images, although we are considering their use as future work, provided that their processing time can be optimised. Therefore, our goal was to develop an image analysis application with the best machine learning algorithm, which is able to establish an objective value of ME using the lowest image resolution possible, in order to optimize either the processing time and/or the requirements of the system employed in the analysis (potentially even providing the possibility of performing through an on-line web application). The evaluated algorithms showed high performance, in terms of image classification. In the training/testing phase, the AUC obtained in all cases was significantly high (>0.98), which was virtually unaffected by the number of pixels used. Only the 1000 pixel data set decreased the AUC of the Keras algorithm. In the trials carried out, it was shown that the AUC for 1000 pixels was more affected by the pixels selected in the random sampling than by the number of pixels used.

Concerning the time spent by each classifier to be trained, it is worth mentioning the outstanding difference between Keras and the remaining algorithms. Furthermore, for each classifier, the time increase was more significant from 50,000 pixels onwards; except with KNN and NB, whose times were barely affected. Thus, between 10,000 and 50,000 pixels, a compromise can be achieved between time spent in training and robustness in the random sampling of pixels.

In the classification step of a real image, KNN and NB were the fastest, regardless of the number of threads. Even in the case of an image with 4 times more pixels than another, the duration was shorter than 1 s. On the other hand, RF and SVM were significantly influenced by both the size of the image and the number of threads involved in the classification. Nevertheless, from six threads on, there was no significant reduction in the time spent classifying the image on the equipment used; thus, it may be unnecessary to invest more computational resources, when the performance is not going to be enhanced substantially.

The global result, when comparing the manually classified and the same automatically classified image with the different classifiers, yielded good results overall. The ratio of positives over the total number of images (i.e., accuracy) was close to 1 in every case. With regard to the sensitivity of Keras, it was the most accurate, with 100% success; while KNN, with 0.844 accuracy, was the most mistaken. The specificity, similar to the accuracy, remained very high for all the classifiers. The main limitation regarding the use of these algorithms was observed in those cases with an extremely high infiltration of fat (70–80%). In those cases, the sensibility of the algorithms to pixel selection was increased, possibly due to the fat infiltration observed within portal and stromal areas. In such cases, alternatives like the use of morphological segmentation algorithms would be helpful to establish accurate values of ME. Another limitation was based on the fact that we did not compare the accuracy of these classification algorithms with other lipid staining methods, such as the oil red procedure; however, our results indicate that the use of specific fat staining procedures, such as Sudan, may be a good choice for the automatic determination of ME in pre-transplant liver biopsies, using minimal requirements with optimal results for those cases with low–average amounts of fat infiltration. Such cases are those in which the pathologist may experience problems in establishing an accurate value of ME.

We conclude, based on these results, that Sudan stain is a suitable and value tool for the evaluation of ME in pre-transplant liver biopsies, as it is suitable for frozen sections, quick, fat-specific, and offers good contrast, which can allow for easy differentiation of fat vacuoles from non-fat vacuoles and unspecific structures; while H & E stain can be used for the evaluation of all other parameters, such as inflammation, infection, necrosis, tumors, and pigment deposits. We propose the introduction of this stain as the technique to use in the evaluation of ME in such biopsies. As our goal was to develop an automatic analytic system which may determine the amount of ME in a stained section, this also may save valuable time for the pathologist, in terms of evaluating the quality of the graft, and minimize (or even eliminate) the possibility of criteria error in the evaluation of this important parameter. Additionally, we developed our application based on the optimization of the quality and the size of the images, in order to optimize the time required for analysis and the computational cost. Regarding the algorithms analyzed, Naïve Bayes and KNN were the best algorithms for the data set on which they were evaluated. Both displayed remarkably high levels of accuracy, sensitivity, and specificity, while also proving to be the fastest in both the training and classification steps, with minimal consumption of hardware resources.

In the future, these algorithms may be implemented in specialized and automatized image analysis applications for liver transplantation, with specific use in Sudan-stained sections.

## 4. Materials and Methods

### 4.1. Liver Samples and Histochemical Procedures

**Figure 5:**
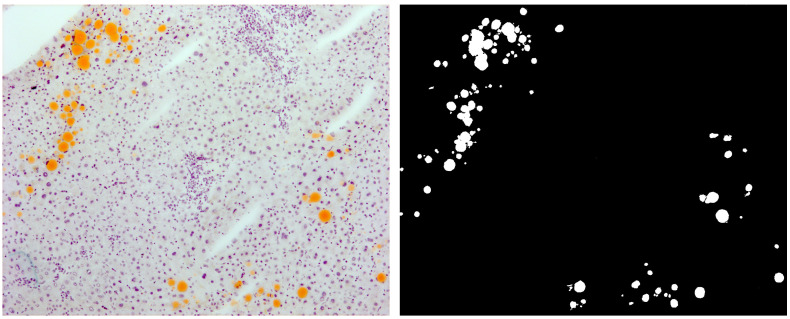
Original image (**left**) and manually classified image (**right**).

Eight micrometer-thick sections were obtained from donor liver samples (n = 20), which were preserved at −80 °C. These sections were stained with an improved Sudan procedure, a specific histochemical fat-staining procedure routinely used in our department to evaluate fat infiltration in frozen samples. Briefly, all samples were sectioned (8 micrometers thick) and stained with a mix of Sudan III and Sudan IV dyes at 50%/50% vol. (Sigma Aldrich, Madrid, Spain) for 10 min at room temperature. The sections were Hematoxylin (Thermo, Barcelona, Spain) counterstained and finally mounted with an aqueous permanent mounting medium. Fat Sudan-positive structures were identified as intracellular orange vacuoles (Figure 5, left). The main artifacts related to the procedure that were detected were air bubbles (due to the aqueous mounting media), sectioning artifacts, unspecific hematoxylin deposits, and unstained blank spaces, mainly due to sinusoid dilation, vessels, and hepatocyte ballonization unrelated to fat deposits.

### 4.2. Imaging

A number of digital images were obtained from stained samples by using a direct-light microscope (Zeiss Axio A10, Carls Zeiss, Jenna, Germany) equipped with a high-quality digital camera (Axio Cam 506, Zeiss) and specialized software (Zeiss Zen ver. 3.0). The technical specifications of the camera are detailed in Table 4 (the complete specifications can be found at https://www.zeiss.com/microscopy/int/products/microscope-cameras.html, accessed on 11 March 2021). Histologically, macrovesicular steatosis is defined as the presence of a large-sized intracellular fat vacuole (with a minimum of two-times bigger than normal nucleus size) which displaces the nucleus to the periphery of the cells [23,24]. To determine the standard diameter of the normal hepatocyte nucleus in frozen sections, we measured 3000 hepatocyte nuclei in frozen sections from 10 healthy livers only stained with hematoxylin, measuring the media of all sizes (73.356 ± 0.177 μm${}^{2}$) and established this value as a standard. Therefore, we considered intracellular macrovesicular fat vacuoles as those with a size ≥146.71 μm${}^{2}$.

### 4.3. Generating Learning Models

To carry out the classification of images, several learning models were generated using different machine learning and deep learning algorithms. Specifically, K-Nearest Neighbors (KNN), Support Vector Machine (SVM), Random Forest (RF), Naïve Bayes (NB), simple Neural Network (NN), and neural network with Tensorflow and Keras [25] (using GPU) were used. Each algorithm, except for Keras, was evaluated with the default parameters, and the image classification process was parallelized. Regarding Keras, a densely connected first layer with 6 nodes with *sigmoid* activation and a final layer with 2 nodes and *softmax* activation were used. *Adamax* was used as the network optimizer. In addition, the computer graphics were configured to use the GPU for training and image classification with this algorithm.

To generate the models, as well as to evaluate their performance, images from optical microscopy of liver tissues at 100, 200, and 400× magnifications were used. Images were obtained with exposure, brightness, and contrast values self-adjusted by the camera software, and images with different levels of adjustment were modified manually. The color histograms of the images were adjusted to the histogram of a reference image defined as the best-fitting by a match-histogram algorithm.

Twenty images with 1920 × 1080 pixel resolution at different magnifications were used. For each image, windows of 10 × 10, 20 × 20, 50 × 50, and 100 × 100 pixels were manually extracted, depending on the enlargement level and size of the vacuoles. A total of 10 50 × 50 windows, 10 100 × 100 windows, 50 20 ×20 windows, and 50 10 × 10 windows were finally taken. The total number of pixels obtained was 200,000. For every pixel, a 6-characteristic vector, defined by RGB and CIE L*a*b color spaces, was obtained:

(1) $$FV_{n}=\left[\begin{array}{cccccc} R_{i} & G_{i} & B_{i} & L_{i} & a_{i} & b_{i}\\ \vdots & \vdots & \vdots & \vdots & \vdots & \vdots \\ R_{n} & G_{n} & B_{n} & L_{n} & a_{n} & b_{n} \end{array}\right]\forall i=1,\ldots ,n,$$

where $FV_{n}$ is the set of feature vectors for all pixels (*n*) used to construct the models, and $R_{i},G_{i},B_{i},L_{i},a_{i},b_{i}$ are the values of the red (R), green (G), blue (B), lightness (L*), green to red (a*), and blue to yellow (b*) channels, respectively, corresponding to the *i*th pixel. This type of feature vector has already been successfully tested in other works related to image analysis [22]. Pixels were tagged with 1 or 0, depending on whether they belonged to a region of the image where there was a fat vacuole (1) or not (0).

Of the total number of pixels, randomized subsets of 100,000, 50,000, 10,000, 5000, and 1000 pixels were selected to train different models with different numbers of pixels. From each pixel subset, 70% were used to train the models, while the remaining 30% were used for testing. The data splitting was carried out by stratified random sampling, in order to obtain a proportionate number of pixels from each class.

Finally, the performance of each algorithm was assessed using the test data sets (30% remaining) of the corresponding training sets. The data splitting, training, and testing processes were executed 10 times with each algorithm and with every data set, in order to determine the average time each algorithm took to train and the average time to classify. Likewise, the AUC of each algorithm for every subset of data was calculated by ROC curves.

### 4.4. Classification Time

With the trained models at 50,000 pixels, the same image was classified at two different resolutions—2752 × 2208 and 1376 × 1104 pixels—in order to determine the time spent by the different models in classifying each image with a different number of threads.

Every performance test was carried out on a laptop with an Intel Core i7-9750H processor at 2.6 GHz, with 6 cores and 12 threads, 16 Gb RAM, and a 4 Gb NVIDIA GTX1620 graphics card.

### 4.5. Classification Validation

The automatically classified images were compared with the same manually classified images by two expert pathologists (Figure 5) through a simple binary image subtraction operation, hence obtaining the TP, FP, TN, and FN values, with respect to the reference image, in order to obtain the overall accuracy, sensitivity, specificity, and precision scores (Equations (2)–(5), respectively). We defined those pixels classified as fat vacuole matching the manually classified image as the TP, those classified as non-vacuole matching the manual image as the TN and, finally, those wrongly classified in the fat vacuole or non-vacuole categories as FP and FN, respectively.

(2) $$Accuracy=\frac{TP+TN}{TP+FP+FN+TN},$$

(3) $$Sensitivity=\frac{TP}{TP+FN},$$

(4) $$Specificity=\frac{TN}{TN+FP},$$

(5) $$Precision=\frac{TP}{TP+FP}.$$

As, in many cases, the fat vacuoles are totally united in the image, to correctly quantify the size and number of vacuoles, it is necessary to have an automatic mechanism that, as far as possible, distinguishes two or more vacuoles together as independent entities. To do this, morphological segmentation has been applied using the watershed algorithm, which has been widely used in the analysis of biomedical and biological images for cell segmentation [26,27,28]. In those cases of extremely high values of ME infiltration (e.g., 70–80%), this classification algorithm experienced some problems in classifying extremely large overlapped vacuoles, although the final result of ME value was not altered due to this limitation.

### 4.6. Web Application Development

In order to assist in the assessment of ME degree, a web application was developed using the *Shiny* [29] framework provided by *R* [30], which enables the rapid development of web applications and simplifies the integration of additional programming languages. The web application was developed in such a way that the user may sequentially follow the steps that lead them from image uploading to the degree of ME quantification.

**Figure 6:**
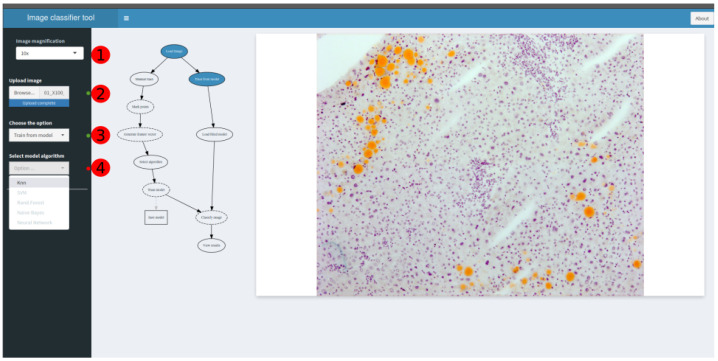
Web application interface image classification steps: (1) Objective magnification selector; (2) Image uploader; (3) Manual or pre-trained model selector; and (4) (if pre-trained) algorithm selector.

**Figure 7:**
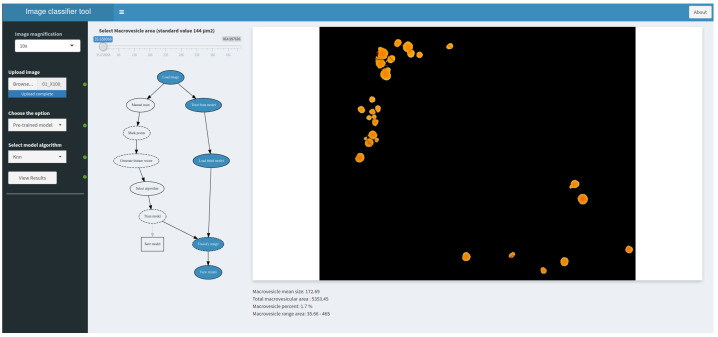
Result of image classification and quantification of fatty vacuoles.

The user sets the number of microscope magnifications when capturing the image (Figure 6(1)). This determines the size of each pixel (in microns). Then, after loading the image (Figure 6(2)), the application allows the user to select a pre-trained model from several algorithms, or to manually train the model by marking the points of interest on the image (Figure 6(3),(4)). Afterwards, the application classifies the image and returns the number and extension of fat vacuoles over the total image, as well as the macrovesicle percentage (Figure 7).

The whole image classification process was conducted in python, mainly using the *scikit-image* [31], *scikit-learn* [32], and *Keras* libraries. Its integration with R was carried out using the R *Reticulate* library [33], which allows for the execution of python code inside R applications.

## 5. Conclusions

Sudan staining is a suitable stain procedure, which can be used in the evaluation of macrovesicular steatosis of the graft in pre-liver transplantation histopathological evaluation. It is a quick and easy technique that, unlike the hematoxylin and eosin stain, is specific to fat identification. Due to its specificity, this stain is suitable for automatic and quantitative evaluation by the use of machine learning algorithms.

The machine learning algorithms Naïve Bayes and KNN showed the best results, in terms of speed and accuracy, in all tests performed for the automatic identification of macrovesicular steatosis in Sudan pre-transplant liver stained sections.

Therefore, the automatic evaluation of macrovesicular steatosis may be performed during the histopathologic evaluation of the quality of the liver graft in the pre-transplant evaluation by using Sudan-stained sections, while other parameters can be established by direct examination of hematoxylin and eosin stained sections by an expert pathologist.

## Figures and Tables

**Table 1:** Average training time and AUC for each algorithm under different numbers of pixels.

Model	Pixels	Average Time	Average AUC	SE Time	SE AUC
KNN	1000	0.000	1.000	0.000	0.000
NB	1000	0.001	1.000	0.000	0.000
NN	1000	0.117	1.000	0.032	0.000
RF	1000	0.118	1.000	0.001	0.000
SVM	1000	0.004	1.000	0.000	0.000
Keras	1000	0.645	0.984	0.022	0.012
KNN	5000	0.000	0.997	0.000	0.000
NB	5000	0.001	0.998	0.000	0.000
NN	5000	0.429	1.000	0.040	0.000
RF	5000	0.207	0.999	0.002	0.000
SVM	5000	0.045	1.000	0.000	0.000
Keras	5000	2.417	0.998	0.113	0.000
KNN	10,000	0.001	0.998	0.000	0.000
NB	10,000	0.001	0.999	0.000	0.000
NN	10,000	0.697	1.000	0.069	0.000
RF	10,000	0.329	0.998	0.002	0.000
SVM	10,000	0.115	1.000	0.001	0.000
Keras	10,000	4.616	0.999	0.080	0.000
KNN	50,000	0.003	0.997	0.000	0.000
NB	50,000	0.005	0.998	0.000	0.000
NN	50,000	1.763	0.999	0.141	0.000
RF	50,000	1.642	0.999	0.011	0.000
SVM	50,000	2.046	0.999	0.011	0.000
Keras	50,000	22.307	0.999	0.333	0.000
KNN	100,000	0.006	0.997	0.000	0.000
NB	100,000	0.011	0.997	0.001	0.000
NN	100,000	2.799	0.999	0.210	0.000
RF	100,000	3.562	0.999	0.079	0.000
SVM	100,000	7.153	0.999	0.107	0.000
Keras	100,000	38.802	0.999	2.507	0.000

**Table 2:** Average classification time (in s), based on number of threads (from 1 to 10) and image size (from 1.5 to 6.1 MB).

Model	Image	1	2	4	6	8	10
KNN	1.5 M	0.09	0.28	0.26	0.24	0.22	0.26
SVM	1.5 M	8.48	4.37	2.41	1.67	1.29	1.46
RF	1.5 M	5.96	3.37	2.01	1.55	1.44	1.47
NB	1.5 M	0.15	0.25	0.22	0.21	0.20	0.21
NN	1.5 M	0.82	0.82	0.73	0.69	0.72	0.57
Keras	1.5 M	40.56	40.56	40.56	40.56	40.56	40.56
KNN	6.1 M	0.32	0.61	0.49	0.45	0.42	0.46
SVM	6.1 M	33.70	17.30	9.28	6.34	4.82	5.39
RF	6.1 M	27.68	13.91	7.85	6.10	5.54	5.77
NB	6.1 M	0.66	0.69	0.55	0.52	0.51	0.51
NN	6.1 M	3.14	2.85	2.61	2.49	2.62	1.94
Keras	6.1 M	163.84	163.84	163.84	163.84	163.84	163.84

**Table 3:** Metrics comparing automatic and manual classification.

Metric	KNN	SVM	RF	NB	NN	Keras
Accuracy	0.996	0.996	0.996	0.997	0.997	0.995
Sensitivity	0.844	0.962	0.956	0.910	0.963	0.972
Specificity	0.999	0.997	0.997	0.999	0.998	0.996
Precision	0.961	0.897	0.894	0.969	0.906	0.856

**Table 4:** Zeiss Axiocam basic specifications.

Sensor Model	Sony ICX 694, EXview HAD CCD II
Sensor pixel count	6 Megapixel. 2752 (H) × 2208 (V)
Pixel size	4.54 μm × 4.54 μm
Exposure time range	250 μs to 60 s.
Spectral sensitivity	Aprox. 400–720 nm. RGB Bayer color filter mask

## Data Availability

Data example is available at https://github.com/MiriamRiquelmeP/Rython.git, accessed on 11 March 2021.

## Sample Availability

Software is available at https://github.com/MiriamRiquelmeP/Rython.git, accessed on 11 March 2021.

## Abbreviations

The following abbreviations are used in this manuscript:

AUC	Area Under the Curve
H&E	Hematoxylin and Eosin
KNN	K-nearest neighbors
ME	Macrovesicular steatosis
NB	Naïve Bayes
NN	Neural Network
RF	Random Forest
ROC	Receiver Operating Characteristic
SVM	Support Vector Machine
